# Design of A Highly Sensitive, Low-cost Underwater Force Plate to Record Substrate Reaction Forces

**DOI:** 10.1093/iob/obae008

**Published:** 2024-03-13

**Authors:** K M Gamel, S Pinti, H C Astley

**Affiliations:** Naval Undersea Warfare Center Division Newport, 1176 Howell St., Newport, RI 002841, USA; Department of Biology at the University of Akron, 302 Buchtel Ave., Akron, OH 44325, US; Department of Biological Sciences Kent State University, 800 E. Summit St, Kent, OH, 44242, US

## Abstract

The study of underwater walking presents major challenges because the small forces applied during underwater walking are difficult to measure due to the lack of a sufficiently sensitive force plate that functions underwater. Understanding the force interaction between the underwater walker and the substrate may lead to better understanding of the evolution, ecology, and biomechanics of underwater walking. The shift from aquatic to terrestrial life was a crucial transition in animal evolution where, underwater walking preceded the invasion of land and combines mechanics from terrestrial locomotion (substrate reaction forces) and aquatic swimming (buoyancy and drag). In this work, we describe our design of a low-cost underwater force plate made using 3D printed multi axis load cells equipped with commercial strain gauges amplified with a custom circuit board, and custom code to gather force data. The use of 3D printed sensors allows customization of the material and thickness of the shear beam load cell to accommodate the loads for a wide range of study species. We show that our design can detect loads as small as 1 mN (filtered) with minimal noise and present sample live animal trials of several species. The 3D multiaxial load cells, circuit design, and custom code are open-source and available online.

## Introduction

Long before the invasion of land, aquatic animals used appendages to generate propulsive force against substrate, in addition to a wide range of animals that still use underwater walking (Edwards 1977; Maude and Williams 1983; Clarac et al. 1987; 1989; Brand 1996; Clack 1996; Martinez et al. 1998; Ashley-Ross et al. 2013; Kawano and Blob 2013; Granatosky et al. 2020). Fish, crustaceans, amphibians, reptiles, and mammals have evolved modified appendages to better interact with the substrate and propel themselves forward (Dickinson *et al*. 2000; Boisvert 2005; Clack 2009; Kawano and Blob 2013; Dickson and Pierce 2019). These morphologies and behaviors provided a starting point for the drastic transformations necessary for life on land (Pierce et al. 2013; Kawano 2014). However, despite the evolutionary importance of underwater walking, the mechanics remain poorly understood due to the lack of substrate force data (Martinez 1996).

The aquatic and terrestrial environments impose different physical forces on the animals moving through them (Dickinson et al. 2000; Ashley-Ross and Bechtel 2004; Ashley-Ross et al. 2009). A swimming animal must generate a thrust force to overcome hydrodynamic drag, and the added mass effect from accelerating (Dickinson et al. 2000). Due to the density of water, buoyancy partially or completely counteracts gravity (Martinez et al. 1998; Calisti et al. 2016). In contrast, air has lower density and viscosity which minimizes buoyancy and drag on land. As a result, terrestrial walking animals must generate propulsive force using ground reaction forces (Wannop et al. 2012; Mcelroy et al. 2014). Without buoyant support, animals must either overcome the force of gravity to raise their body above the substrate or overcome surface friction from belly drag (Kawano and Blob 2013). In underwater walking, propulsive force is generated via substrate interactions, creating a ground reaction force like terrestrial walking (Kawano and Blob 2013). However, like swimming (Lauder 2015), the animal must overcome hydrodynamic drag and the added mass effect (Maude and Williams 1983; Martinez 2001; Lim and DeMont 2009). Buoyancy partially counteracts gravity, as seen in swimming, which greatly reduces the load limbs encounter during substrate interaction (Martinez 1996). Adjusting the density of the body by changing internal gas volume can allow the animal to sink to the bottom and interact with the substrate (Zug and Arbor 1971; Peterson and Gomez 2008). This substrate interaction creates a unique mix of forces, with propulsion via substrate reaction forces (SRF) as the key link between terrestrial walking and underwater walking.

Despite the importance of substrate forces in underwater walking, the fundamental kinetics have been difficult to measure due to the lack of a sufficiently sensitive measuring system, the risk and difficulty of waterproofing equipment, and the complexity of hydrodynamic forces in aquatic environments. Researchers have previously tried to overcome these difficulties in various ways but resulted in difficult methodological approach. One used a calibrated gelatin-based substratum to gather total forces from flying gurnards (*Chelidonichthys lucerna*) (Jamon et al. 2011). This study successfully detected SRF, but only the overall magnitude of the forces could be quantified, and the force direction could not be resolved (Jamon et al. 2011). Two prior studies report single leg forces in crustaceans (Clarac and Cruse 1982; Klarner and Barnes 1986). One linked force data with neurological data of a rock lobster (*Jasus lalandii*, 3.9 N body weight) using a force transducer attached to the fourth or fifth limb perpendicular to the ground (Clarac and Cruse 1982). The attachment allowed recording of a range of forces seen during aquatic locomotion but may alter the kinematics and the substrate/limb engagement (Clarac and Cruse 1982). Furthermore, because the sensor moved with the limb, the forces components are relative to the limb segment not the body direction of motion (Clarac and Cruse 1982). Another study used a small platform with two load cells (vertical and one horizontal axis) only big enough to gather single leg forces of a crayfish (Klarner and Barnes 1986). Evidence showed that each pair leg was under different load, where the third pair exerted the largest vertical force (Klarner and Barnes 1986). However, because each trial only gathered a subset of force components, authors had to combine multiple trials to gain a complete understanding of SRF (Klarner and Barnes 1986). Single leg forces allow calculation of joint moments, while full body kinetics reveal the effect of the environment and SRF on the whole body, in addition to revealing the energetics needed to perform underwater walking. Even with these insightful results, replication of these methods has been extremely problematic, due to difficulties waterproofing sensors and the need for extreme sensitivity, leaving major gaps in our knowledge of underwater walking.

To enable collection of detailed SRF data, we developed an underwater force plate with the specific aim of making it accessible for other researchers to recreate. While other researchers have built highly sensitive force plates before (Blickhan and Full 1993, Zumwalt et al. 2005, Hsieh 2010), this system was designed primarily to function underwater and uses lower cost components. The independent researcher can alter material and design stiffness of the 3D printed load cells, creating a force plate for their species of choice, then apply strain gauges with our custom circuit board to power and amplify the electrical signal to gather SRFs. Gathering detailed SRFs of underwater walking will fill critical transitional links in the evolutionary transition from water to land and help compare adaptation for underwater walking (Koehl 1996; Martinez et al. 1998; Dickinson et al. 2000). We show this system is capable of detecting underwater walking SRFs and hydrodynamic wakes. A full description and analysis of animal data will be published in the companion paper (Gamel et al. 2024).

## Methods

### Force plate

**Fig. 1:**
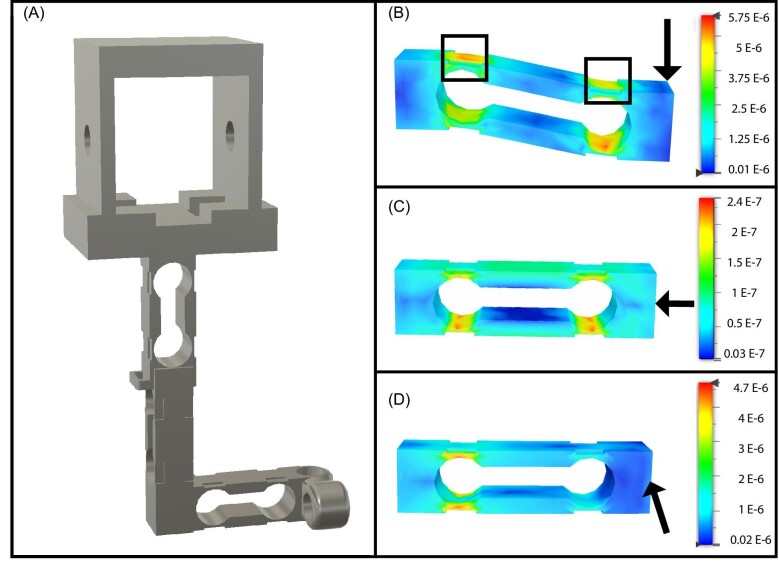
Load cell design. (**A**) A corner sensor with 3 shear beam load cells arranged to measure forces in the *XYZ* axes. (B–D) are finite element models simulated in Fusion 360 to test strain under static loading. The left side is fixed and loads are applied as indicated by the arrows on the right. Color bars shows strain (unitless), and deformation is exaggerated in the images. (B) A single, vertical load cell under 0.5 N of force in the vertical direction. The black boxes represent the ideal locations to place the strain gauges where deformation is amplified by structural design. (C) The load cell under load of 0.5 N applied from right to left. (D) The load cell under a load of 0.5 N applied perpendicular to the plane of the image. Note that in (C) and (D), deformation is minimal at the suggested strain gauge placement locations in (B).

**Fig. 2:**
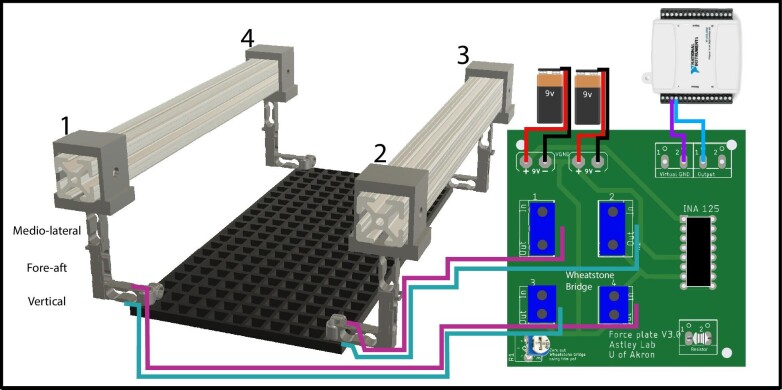
A diagram of the fully constructed force plate with vertical, fore-aft, medio-lateral load cells. The diagram depicts the strain gauge physical placement for the front pair of vertical load cells, configured in a full Wheatstone Bridge. Other channels are similar, but omitted for clarity; complete wiring instructions are available on GitHub. (KaeGamel/Underwater-Force-Plate: Underwater Force Plate [github.com]).

To create a sufficiently sensitive force plate, we designed a single-point shear beam load cell (Fig. 1A) in Autodesk Fusion 360 (AUTODESK, San Francisco, CA, USA). This shape allowed for the material to deform at the point where the material is thinnest when loaded in a cantilever configuration (Fig. 1B), while also minimizing deformation in the opposing directions, which reduced the mechanical crosstalk (Fig. 1C–D). On each load cell, four or two strain gauges (KFH-3–350-C1-11L1M2R, Omega Engineering, Inc, Norwalk, CT, USA) were placed over the thinnest regions (Fig. 1B) to sense material deformation. The first version of this system used four strain gauges, but subsequent versions used only two, which reduced cost and troubleshooting difficulty without the loss of sensitivity. These strain gauges were arranged in a Wheatstone bridge configuration and connected to an INA 125P amplifier via a custom circuit (Fig. 2). This amplifier both provided regulated power (5 volts) to the Wheatstone bridge and amplified this signal. The maximum output range of each amplifier circuit was 9 to −9 V but was typically in the range of −3 to +3 V during experiments.

Most force plates use strain gauges attached to similar metal load cells to gather force data due to the high forces during terrestrial locomotion (Heglund et al. 1982; Biewener and Full 1992; Dai et al. 2011). However, the high stiffness of metal limits the ability to sense smaller forces. Because the forces applied by underwater walkers are predicted to be very small due to buoyant support, a force plate to gather such small loads needs lower stiffness to deform sufficiently to be detectable. This force plate uses 3D printed material formed from a polymer-based resin, which has lower stiffness and thus allows smaller forces to cause detectable strain on the material, similar to a highly sensitive terrestrial force plate (Reinhardt and Blickhan 2014). Using a resin-based SLA printer (Formlab 2) permits material uniformity and access to a strong, stiff plastic material (Tough 2000 resin, tensile strength = 2.2 GPa, flexural strength = 65 MPa, flexural modulus = 1.9 GPa, and elongation at break = 48%). This material was sufficiently compliant but did not plastically deform under typical loads in our system. If irregularities were seen in the 3D printed part, we deemed it flawed and discarded, only using ideal pieces. We determined the natural frequency of the force plate by dropping a 10 g weight while underwater and examining the resulting oscillations of the voltage signals.

To ensure our force plate had a large sensing area but would not be damaged by torques induced by off-center loads, we used a platform connecting four instrumented corners, with three orthogonal load cells per corner to measure forces in each axis (Fig. 2) (Biewener and Full 1992). We powered our force plate using eight, 9 V batteries, two for each circuit board as this was found to reduce noise compared to using AC and DC converters. Three load cells were oriented to gather forces in the vertical (*Z*), fore-aft (*X*), and medio-lateral (*Y*) axes (Fig. 1A). Threaded holes were used to attach a platform/substrate and a clamping mechanism anchored the four corner load cells together using stainless steel beams. The platform was a plastic egg crate material, where square spaces were 10 mm by 10 mm, allowing water to flow through the sensing surface area. Later designs incorporate an anchoring structure using 80–20 T-slot aluminum beams (Fig. 1A). This anchoring type allows for ease of adjustments of the corner pieces for differently sized sensing areas, the ability to dismantle for travel, and adaptability for future users.

Aquarium silicone was used to completely waterproof the electrical components of the force plate that are exposed to water. Prior to attaching strain gauges to load cells, to prevent corrosion from damaging the system, we placed silicone overtop any wire junctions (changes in wire size) and then applied heat shrink to form a complete seal. After the strain gauges were affixed to the 3D printed load cells with cyanoacrylate glue, silicone was applied on top of the strain gauge, leaving no exposed wire. No variability of the electrical signal was seen due to varying amounts of silicone applied. Due to the importance of silicone protection to various parts of this force plate, periodic inspection and repair of the silicone is recommended. Subsequent iterations of the force plate design found applying M-Coat A polyurethane (Micro Measurements Inc., Raleigh, NC, USA) followed by silicone to be the most optimal waterproofing technique.

### Data acquisition

**Fig. 3:**
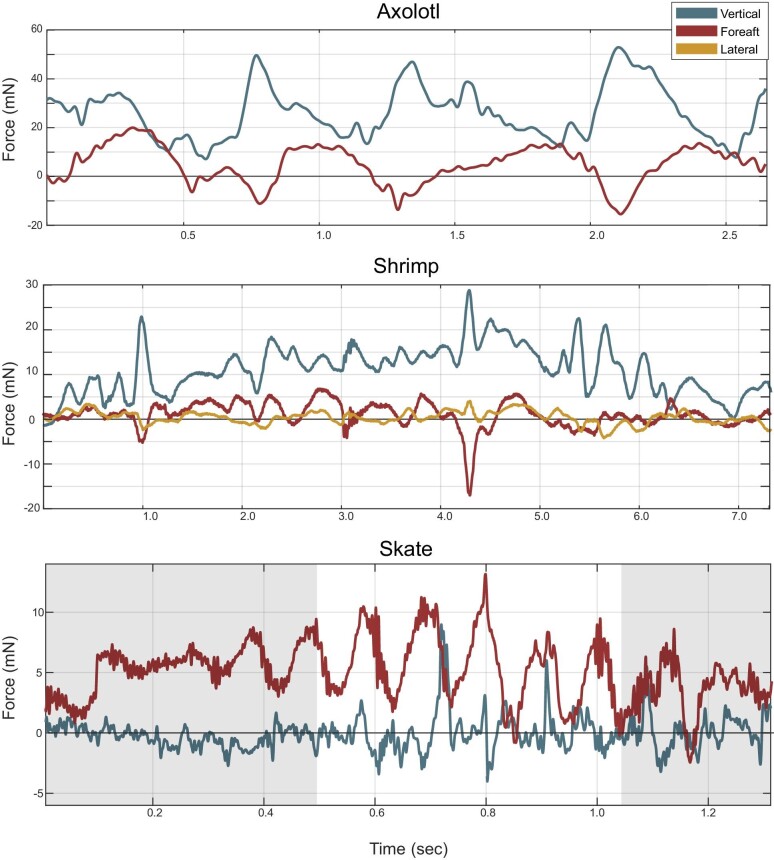
Example data from animals crossing the underwater force plate. In all graphs, blue is the vertical force, red is the fore-aft force, and yellow (when present) is the lateral force. Propulsive fore-aft forces are positive, while braking force is negative. (A) An axolotl (*Ambystoma mexicanium*) performing underwater walking. (B) A prawn (*Pandalus platyceros*) performing underwater walking. The braking force at 4.2 s indicates a “stumble.” (C) A skate (*Beringraja bioculata*) swimming above the modified force plate in still water. Highlighted in white shows when the body of the skate is fully over the force plate. The skate slightly touches the force plate at approximately 0.8 seconds, resulting in a relatively larger vertical force.

We used a National Instruments Data Acquisition (NIDAQ) USB-6002 (National Instruments Corporation, Austin, TX, USA) and NIDAQ Tools MX package to gather data with MATLAB (MathWorks, Natick, MA, USA), or IGOR (WaveMetrics, Portland, OR, USA). This NIDAQ was configured in differential input mode, which allowed for individual grounds from each circuit, thus we used the amplified output and virtual ground wired to +/− screw terminals, respectively. The data acquisition (DAQ) function within each program gathers signals from two vertical channels, one fore-aft, and one medio-lateral. Vertical outputs were instrumented with two channels/circuit boards because the platform is in the vertical plane and amplification must be equal at the front and back of the force plate. The vertical output is equal to the sum voltage of both vertical channels (the front and back channels). For more detail, reference book chapter “Force platform and kinematic analysis” from the Biomechanics Structure and Systems: A Practical Approach (Biewener and Full 1992). Early versions of the force plate used similar configuration for fore-aft and lateral channels, but we found that we could unify these two channels without loss of sensitivity. We use a low pass filter with rejections starting at 10 Hz and complete rejection over 20 Hz (Fig. 3), meaning that the attenuation of signals above 10 Hz increases with frequency until 20 Hz. The scripts are published on GitHub (KaeGamel/Underwater-Force-Plate: Underwater Force Plate [github.com]).

### Calibrations

To perform calibrations, we used 6 masses within the predicted range of forces (±10, ±5, and ±2 g of mass equal to ±98.06, ±49.03, and ±19.61 mN, respectively). Calibrations were done out of water and followed the procedures of protocol 1 in Biewener and Full (1992). We applied the series of weights in all six directions, ±*X*, ±*Y*, and ±*Z*. We attached a Kevlar to the middle of the force plate and used it as a cable to pass over a pully at the same height as the force plate. The weights were added and removed 5–8 times over a 30 s duration. The voltage change was recorded for each circuit for each directional load. We then placed the force plate in water to compare the voltage change with our dry calibrations. Due to buoyancy, the voltage change was 82% of what was seen out of water, which was correct considering buoyant support on steel weights. We then used the scipy.linalg.lstsq function in Python (Python, Lacombe, LA, USA), to compute the 3 × 3 calibration matrix which transforms the voltage changes to the calibration loads as shown below. To assess the accuracy and precision of the matrix, we multiplied this matrix by the calibration voltages and computed the difference between the calculated and known calibration weights.


\begin{eqnarray*}
\frac{{{\mathrm{Voltage}}\,\,{\mathrm{Output}}}}{{\begin{array}{@{}*{1}{c}@{}} {{\mathrm{Vertical}}\,\,{\mathrm{Voltage}}}\\ {{\mathrm{Fore \!-\! aft}}\,\,{\mathrm{Voltage}}}\\ {{\mathrm{Lateral}}\,\,{\mathrm{Voltage}}} \end{array}}} &&\times \left[ {\begin{array}{@{}*{3}{c}@{}} {107.11,}&\quad{ - 1.17}&\quad{8.82}\\ { - 4.04,}&\quad{134.16,}&\quad{ - 1.18}\\ { - 3.57,}&\quad{0.819,}&\quad{173.75} \end{array}} \right]\nonumber\\
&&\quad = \frac{{{\mathrm{Calibrated}}\,\,{\mathrm{Force}}}}{{\begin{array}{@{}*{1}{c}@{}} {{\mathrm{Vertical}}\,\,{\mathrm{Force}}}\\ {{\mathrm{Fore \!-\! aft}}\,\,{\mathrm{Force}}}\\ {{\mathrm{Lateral}}\,\,{\mathrm{Force}}} \end{array}}}
\end{eqnarray*}

### Experimental animals

We tested the force plate with a functionally diverse set of three species: (1) The axolotls (*Ambystoma mexicanium*), fully aquatic salamander; (2) The big skate (*Beringraja binoculata*), a Chondrichthyan; and (3) the California spot prawn (*Pandalus platyceros)*, a crustacean.

Five axolotls were purchased from the Ambystoma genetic stock center. All axolotls were adults (mean ± s.d., total length = 25.6 ± 1.6 cm, underwater weight = 14.2 ± 11.8 mN, and mass = 112.68 ± 10.8 g). Axolotl procedures were approved under University of Akron IACUC protocol 22–05-04-AAC. Two skates (pectoral width approximately 10 cm, length without tail approximately 16 cm) were housed at Friday Harbor Laboratories in a 1000-gallon plastic stock tank at 16°C in salt water. Six prawn (mass = 20.3 ± 1.6 g, length = 13.3 ± 0.3 cm) were trolled in the Salish sea and stored in a 55-gallon tank. Skate and Prawn procedures were approved under University of Washington IACUC- 4308–4.

## Results and discussion

The underwater force plate was capable of detecting forces as low as 5 mN in raw data, but once filtered, can detect forces as low as 1 mN. The noise when unloaded has an amplitude of 12 mV, with frequency spikes at 60 Hz and harmonics thereof from room ambient electrical power (Supplementary Fig. S1). The natural frequency of the force plate in water is 80 Hz and is more than an order of magnitude greater than the animal footfalls (>2 Hz) (Kawano and Blob 2013). Approximately 2 V difference generally occurred in the vertical cannels after the system was placed in water and turned on, but once zeroed and stabilized, further drift rarely occurred during trials (less than 0.1 V over the duration of 60 s trial). If visual drift occurred, it could be corrected for in MATLAB using a linear interpolation using the output before and after the animal was in contact with the force plate. A weight was placed on the force plate before or after every trial to ensure proper function and to synchronize digitized kinematic data and force data. The difference between calculated and known calibration loads was +−2.3 mN and the *r*² value of the matrix was 0.993, indicating high linearity and a good fit. Additionally, we found that when there is no force applied in a given direction, the calibration matrix gives an average value of 0.94 mN, showing that loads greater than 1 mN are most likely genuine. We had substantial difficulty calibrating at the lowest masses due to the small size of the masses attached to Kevlar string, so we isolated a single shear beam load cell. A single shear beam load cell setup was used to ensure 2 mN of force was attainable and gathered voltage from the applied mass of ±0.2, ±0.5, ±1, ±2, ±5, and ±10 g five times each weight (*r*² value of 0.95). We present three examples of reaction force data: an axolotl walking across the force plate (showing vertical and fore-aft force), a prawn walking across the force plate, and a juvenile skate swimming just above the force plate (Fig. 3). We detected SRF from the axolotl and the prawn and detected hydrodynamic forces from the skate swimming over the force plate.

**Fig. 4:**
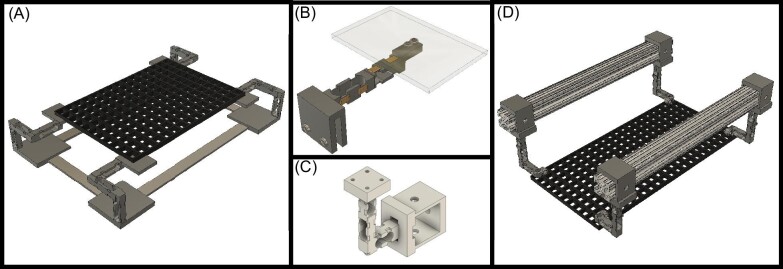
Different iterations of the force plate design. (A) is the first design of the full set up force plate with 4 corners each set up with 3 shear beam load cells with 4 strain gauges on the load cell. (B) Small force plate with 2 single shear beam load cells with 4 strain gauges on each load cell. (C) Even smaller force plate with 3 single shear beam load cells with 2 strain gauges on each load cell. This could be for single footfalls or smaller jumping species (Bartlett et al. in review). (D) is the first design of the full set up force plate with 4 corners each set up with 3 shear beam load cells with 2 strain gauges on the load cell.

This force plate can be varied as needed for different circumstances and diverse species. Our force plate uses, four corners of 3 uniaxial load cells (Fig. 4A, D), where for smaller sensing areas, one trio of load cells can support a small platform. During preliminary trials, a 3.81 by 3.81 cm platform was created with only 2 load cells, with 4 strain gauges on each load cell, used for measuring SRFs of small (∼6 cm long) fish during amphibious movements (Fig. 4B, C). This design was selected to concentrate and amplify the force on a single attachment point instead of distributing load across four corners. Larger platforms used for larger species, in size and weight, require multiple supports to prevent damage due to the potential magnitude of torques from off-axis loading. Additionally, thicker walled load cells with four cornered force plate can be used for bigger species. An extreme example of high force configuration are the S-shaped uniaxial load cells made of the same 3D printed material and with the same circuitry and amplifier system used in a burrowing robot in our lab to detect mechanical forces over 150 N (Edwards, personal observation). This robot presents an example of how modification to the 3D print can collect other types of forces to determine work needed of a system. Each 3D printed design is set up to measure directional forces and depending on methodology, either pure tension system (S-beam load cell) or tension and compression (shear beam load cell) can be used. Depending upon the needs and design constraints of the system, a wide range of load cells can be fitted with the system described above, including S-beam load cells for measuring forces along the axis of the load cell or shear beam load cells for measuring forces perpendicular to the long axis of the load cell.

We have attempted to make this system an accessible as possible to other researchers and include detailed instructions in the GitHub site. The 3D printed load cells and use of widely available electronic components reduce the overall cost of the system (~800$ USD) and increase accessibility. In addition to the force plates at University of Akron, we have constructed one at Friday Harbor Labs at the University of Washington, and provided modified versions to researchers at New Jersey Institute of Technology and Clemson University. As a test of the accessibility of this system, researchers at Ottowa University successfully built a dual terrestrial/aquatic version of the force plate with minimal guidance using the tools on GitHub (Donatelli and Standen, pers. comm.).

The development of an extremely sensitive, low cost, and customizable underwater force plate will allow a more complete understanding of the biomechanics of underwater walking, shedding light on the water to land transition. This mechanical information in turn will lead to a better understanding of why animals interact with the substrate, the benefits and cost, and the control and dynamics of these behaviors. The low-cost, open-source nature of this system will increase accessibility, particularly for resource-limited labs, for both this specific use case (underwater force plate) and subsequent modifications for more general force measurements.

## Supplementary Material

obae008_Supplemental_Files
